# A controlled trial of intra-articular corticosteroids with or without methotrexate in oligoarticular juvenile idiopathic arthritis

**DOI:** 10.1186/1546-0096-12-S1-P1

**Published:** 2014-09-17

**Authors:** GP Bracciolini, S Davì, A Pistorio, A Consolaro, S Verazza, B Lattanzi, G Filocamo, S Dalprà, M Gattinara, V Gerloni, A Insalaco, F De Benedetti, A Civino, G Presta, L Breda, L Lepore, C Maggio, F Garofalo, S Magni-Manzoni, D Rigante, A Buoncompagni, M Gattorno, C Malattia, P Picco, S Viola, N Ruperto, A Martini, A Ravelli

**Affiliations:** 1 Istituto Giannina Gaslini, Genoa, Italy; 2 Istituto ortopedico G. Pini, Milan, Italy; 3 Ospedale Pediatrico Bambino Gesù, Rome, Italy; 4 Ospedale Cardinale G. Panico, Tricase, Italy; 5 Ospedale Policlinico, Chieti, Italy; 6 Istituto Burlo Garofolo, Trieste, Italy; 7 University of Palermo, Palermo, Italy; 8 Ospedale degli Infermi, Biella, Italy; 9 Policlinico A. Gemelli, Rome, Italy; 10 University of Genoa, Genoa, Italy

## Introduction

In contrast with the numerous controlled trials conducted in polyarticular or systemic juvenile idiopathic arthritis (JIA), little evidence-based information is available for oligoarticular JIA. As a result, the management of children with this subtype, which is the most prevalent in Western countries, is largely empiric. Intra-articular corticosteroid (IAC) injection is the therapy of first choice for oligoarthritis in many pediatric rheumatology centers. However, although IAC injections are usually highly efficacious, relapses of synovitis are common and sometimes occur only a few months after the procedure. It is still unclear whether concomitant administration of methotrexate (MTX) may increase and prolong the effectiveness of IAC injections.

## Objectives

To compare the efficacy of IAC injection administered as monotherapy or in association with MTX in children with oligoarticular JIA in a phase II, randomized, actively controlled, multicenter trial.

## Methods

Inclusion criteria were oligoarticular JIA by ILAR criteria, age < 18 years, and parent informed consent. Patients who were previously treated with synthetic or biologic DMARDs, had undergone an IAC injection < 3 months, were newly injected only in 1 knee, or had active uveitis were excluded. Patients enrolled were randomized 1:1 to receive either IAC therapy alone (Arm 1) or IAC therapy plus MTX (Arm 2). MTX was given orally at 15 mg/m2/week (maximum 20 mg/week). All patients were followed for 12 months and were assessed at 3, 6 and 12 months. The primary outcome was synovitis flare, defined as recurrence, persistence or new onset of synovitis in 1 or more injected or uninjected (i.e. previously unaffected) joints. Flare rate/probability was compared by chi-square and Kaplan-Meier methods.

## Results

A total of 207 patients (50 boys and 157 girls) were enrolled, 102 in Arm 1 and 105 in Arm 2. Fifteen patients lost to follow-up <6 months were included only in the intention-to-treat (ITT) cohort. Patients in arms 1 and 2 were comparable for demographic features and median number of injected joints (2 vs. 2). In the ITT cohort (n=207) flare of synovitis occurred in 133 patients (64.2%), 69 (67.6%) in Arm 1 and 64 (60.9%) in Arm 2 (p=0.31), whereas in the as-observed (AO) cohort (n=192) flare of synovitis occurred in 118 patients (61.4%), 64 (66%) in Arm 1 and 54 (56.8%) in Arm 2 (p=0.19). By Kaplan-Meier analysis, the probability of synovitis flare in the 2 treatment arms was comparable in both ITT and AO cohorts (log-Rank test: p=0.18 and 0.07, respectively). However, among the 118 patients who flared in the AO cohort, flare in injected joints occurred more frequently in Arm 1 than in Arm 2 (46/64, 71.9% vs. 29/54, 53.7%; p= 0.04).

## Conclusion

The association of oral MTX did not increase the overall effectiveness of IAC therapy. However, flare of synovitis in injected joints occurred less frequently in patients who received concomitant MTX.

## Trial registration identifying number

FARM7Y279L, AIFA, Italy.

## Disclosure of interest

None declared.

